# A retrospective audit of nursing-related morbidity recorded in a state hospital in KwaZulu-Natal

**DOI:** 10.4102/curationis.v42i1.1969

**Published:** 2019-03-27

**Authors:** Spumelelo P. Nyide, Petra Brysiewicz, John Bruce, Damian L. Clarke

**Affiliations:** 1 School of Nursing and Public Health, University of KwaZulu-Natal, Durban, South Africa; 2 Pietermaritzburg Metropolitan Trauma Service, Department of Surgery, University of KwaZulu-Natal, Pietermaritzburg, South Africa

## Abstract

**Background:**

Health care professionals are expected to deliver safe and effective health services; however there is increased realisation that adverse events in the health system are a major cause of preventable morbidity and mortality.

**Objectives:**

To conduct a retrospective audit of nursing-related morbidities in a state hospital in KwaZulu-Natal, South Africa.

**Method:**

A retrospective audit of nursing-related morbidities documented by the surgical service was carried out using the Hybrid Electronic Medical Registry data for a period of 3 years – 01 November 2013 to 31 October 2016.

**Results:**

There were a total of 12 444 admissions to surgical service during the study period, with 461 nursing-related morbidities reported. There was an increase in the number of documented nursing-related morbidities noted during November 2015 to October 2016, with 79% of all reported nursing-related morbidities documented during this period. A total of 54% of nursing-related morbidities were associated with males (*n* = 248) and 46% (*n* = 213) with females. The most commonly documented nursing-related morbidity was drugs/medication (*n* = 167, 36%) with the second most common being adjunct management (*n* = 130, 28%).

**Conclusion:**

The study has identified the most commonly documented nursing-related morbidities in the surgical service of a state hospital. The findings of the study could provide direction for further research and educational initiatives.

## Introduction and background

There is increased realisation that adverse events in the health system are a major cause of preventable morbidity and mortality and that these adverse events are often associated with human error (Duarte et al. 2015). This has led to greater emphasis being placed on the recognition of adverse events and on systematic efforts to prevent and reduce the incidence and impact of these adverse events on patients. Patient safety is one of the important concepts in the field of health care globally and a key factor in ensuring provision of quality health care (Kong & Clarke 2016). Health care is expected to be delivered safely and effectively by health care professionals. Wilson et al. (2012) state that harm to patients, which could be prevented, resulting from erroneous health care is unacceptable. Adverse events owing to medical errors represent a major source of morbidity and mortality worldwide and approximately 10% of all patients admitted to hospital suffer adverse events (Jha et al. 2013). Reid-Searl, Moxham and Happell (2010) estimate that approximately one in six patients experience an adverse event as an unintended consequence of receiving care in an Australian hospital.

In South Africa, only a few studies have been undertaken to quantify the medical error rates (Welzel 2012). The only recent study of medical error rate which includes South Africa was conducted in developing countries and revealed that 8.2% of admissions suffered medical errors (Wilson et al. 2012). However, the authors highlighted that there was a great possibility of undercounting of errors in the study. Errors do not only affect the patients’ outcome but are also financially costly to the health care system. Pepper and Slabbert (2011) state that there is a rapid increase in the number and size of claims which may indicate that South Africa is on the verge of a medical malpractice litigation storm. According to the Medical Practice Society (MPS), the cost of reported claims in South Africa has more than doubled (Malherbe 2013). The claims exceeding R1 million have increased by nearly 550% compared with those of 10 years ago, while claims valued at over R5 million have increased by 900% in the past 5 years (Malherbe 2013). In March 2015, the South African Department of Health held a medico-legal summit to discuss solutions to the rapidly growing crisis of medico-legal claims, referring to this phenomenon as an explosion of medical malpractice litigation. In 2016, the Minister of Health assigned a ministerial task team to lead the way in addressing medico-legal litigation and this was followed by a medical malpractice workshop in 2017. The mandate of all of these initiatives was to find a solution to the medico-legal claims that continue to threaten the South African health care system (SALRC 2017).

A variety of strategies have been introduced to improve patient safety and eliminate harm owing to errors in health care provision. Despite this, human error and adverse events continue to impact negatively on patient outcomes (Chassin & Loeb 2013). All health care workers need to be involved in efforts to counter the impact of human error on adverse patient outcomes.

The biggest single work force in the health system is the nursing staff and their role in countering and reducing adverse events is central. Nursing-related morbidities are a long-standing health care phenomenon that dates back to the inception of the nursing profession in 1859 when Florence Nightingale attended the Crimean War in Turkey (Schmalbach 2015). There is however little literature on the contribution of nursing staff to adverse patient events and on the role of nursing-related error in adverse events. This paucity of literature is especially marked in low- and middle-income countries. In light of this, we set out to review all nursing-related morbidity recorded in surgical patients in a major South Africa teaching hospital.

## Aim

To conduct a retrospective audit of nursing-related morbidities documented by the surgical service of a state hospital in KwaZulu-Natal (KZN), South Africa.

## Methods

This study was conducted using a descriptive, non-experimental quantitative design based on a retrospective audit of nursing-related morbidities that were documented by the surgical service from November 2013 to 31 October 2016.

### Setting

The research setting was the surgical department at a tertiary hospital in the largest city in the interior of the KZN province. This hospital is a major teaching centre that offers tertiary services to a high number of surgical patients in the western third of KZN and serves a population of approximately 3.5 million people (including at least 19 other district hospitals). The hospital has 530 commissioned beds and is presently using 507 beds of which 81 beds are dedicated to the surgical department, accounting for 16% of the total available beds. Compared to other departments, a high number of beds are dedicated to the surgical department because of the high volume of surgical patients.

### Population and sampling

The study population comprised all patients admitted to the surgical service in the state hospital, for both elective and emergency reasons, between 01 November 2013 and 31 October 2016. Total population sampling was used to collect information compiled by the surgical service of all surgical patients who sustained nursing-related morbidities. Paediatric data were excluded owing to poor documentation and underreporting of nursing-related morbidities.

### Data collection process

The Pietermaritzburg Metropolitan Trauma Service (PMTS) has developed a Hybrid Electronic Medical Registry (HEMR) which captures data on all surgical patients. The HEMR also has a separate component which records all surgical morbidity and mortality (including both nurse- and doctor-related morbidity). Following ethical approval and hospital permission, the nursing-related morbidity data for adult patients were extracted from this registry for the 3-year study period. The nursing-related morbidity data were then further categorised into six different areas (Box 1).

BOX 1 Categorisation of nursing-related morbidities.

Adjunct management

- Intravenous lines errors – for example, central and peripheral lines left open, peripheral line infiltrations
- Intercostal drains errors – for example, accidental disconnection of ICD bottle, poor documentation of output and kinking of the tubes
- Gastric tubes errors – for example, nasogastric tubes, nasojejunal tubes not flushed and blocked; percutaneous endoscopic tube removed accidentally

Wound management

- Wound dressings omission
- Poor stoma care – for example, surrounding skin excoriation, leakage from colostomy to a laparotomy wound causing sepsis
- Non-portable vacuum drain errors – for example, poor documentation of vacuum output and poor vacuum dressing care, resulting in blockage

Patient management

- Bedsores – for example, poor pressure part care
- Falls
- Restraints errors

Resuscitation

- CPR errors – for example, ineffective CPR and not initiating CPR
- Errors in using resuscitation equipment – for example, failing to check and use equipment in advance and during resuscitation

Vital signs

- Vital signs omission
- Abnormal vital signs not reacted upon

Drug errors

- Drug overdosing
- Drug omission

ICD, intercostal drains; CPR, cardiopulmonary resuscitation.

### Data analysis

The collected data were analysed using SPSS 22 with the assistance of a statistician. Descriptive statistics based on percentages and frequency of distribution were used to describe and summarise the data. Inferential statistics were carried out using Chi-square test (significance was set as *p* < 0.5).

## Ethical consideration

Ethical approval to maintain the HEMR system has been obtained from the Biomedical Research Ethics Committee (BCA221/13 BREC) of the University of KwaZulu-Natal and from Research Unit of the Department of Health, South Africa. Ethical approval was also obtained for this study from the Biomedical Research Ethics Committee of the University of KwaZulu-Natal (BREC Ref no. 481/16). This study was conducted with the approval of the HEMR system research group. This study is considered to have no apparent risk to the subjects as it does not identify subjects but it audited the data already entered into the HEMR system for nursing-related morbidities only. The data for this project were extracted from this system and then saved onto a memory stick which was kept in a secure location only accessible to the research team.

## Results

### Overview of nursing-related morbidity

There were a total of 12 444 admissions to surgical services during the study period (November 2013 – October 2016), with 461 nursing-related morbidities reported. This gives an incidence rate of 3.7%. There was an increase in the number of documented nursing-related morbidities noted during the last year of the study period (November 2015 – October 2016), with 79% of all reported nursing-related morbidities documented during this period. The surgical service is made up of two disciplines, namely general surgery and trauma (Tables 1 and 2). The majority of the patients experiencing nursing-related morbidities were general surgery patients (Table 3). The frequency of the nursing-related morbidities by gender differences showed that a total of 54% of nursing-related morbidities were associated with males (*n* = 248) and 46% with females (*n* = 213) (Table 4). The age of patients ranged from 15 years to 93 years and the mean age was 45 years (Table 5).

**TABLE 1:** Nursing-related morbidities per year.

Period	Total admissions to surgical service	Nursing-related morbidities	
		Frequency (*n*)	Percentage
2013 and 2014	4360	44	1
2014 and 2015	3842	5	1
2015 and 2016	4242	366	9
**Total**	**12 444**	**461**	**4**

**TABLE 2:** Nursing-related morbidities by discipline.

Discipline	Study period in years								Admissions
	Total morbidities		2013 and 2014		2014 and 2015		2015 and 2016		
	*n*	%	*n*	%	*n*	%	*n*	%
Trauma	166	4	21	13	16	9	129	78	4800
Surgery	295	4	23	8	35	12	237	80	7644

**TABLE 3:** Categories of nursing-related morbidities by discipline.

Nursing-related morbidities	Trauma morbidities (*n* = 166)		General surgery morbidities (*n* = 295)		Total	
	*n*	%	*n*	%	*n*	%
Adjunct management	70	42	60	20	130	28
Wound management	27	16	46	16	73	16
Patient management	13	8	17	6	30	7
Resuscitation	0	0	4	1	4	1
Vital signs	6	4	51	17	57	1
Drugs/medication	50	30	117	40	167	36
**Totals**	**166**	**3.5**	**295**	**3.7**	**461**	**100**

**TABLE 4:** Nursing-related morbidities per category per years.

Nursing-related morbidities category	Gender				Years						Total	
	Females		Males		2013 and 2014		2014 and 2015		2015 and 2016			
	*n*	%	*n*	%	*n*	%	*n*	%	*n*	%	*n*	%
Adjunct management	45	21	85	34	7	16	10	19.5	113	31	130	28
Wound management	31	15	42	17	3	7	10	19.5	60	16	73	16
Patient management	12	6	18	7	2	4	2	4.0	26	7	30	7
Resuscitation	2	1	2	1	0	0	0	0.0	4	1	4	1
Vital signs	46	21	11	5	4	9	9	18.0	44	12	57	12
Drugs/medication	77	36	90	36	28	64	20	39.0	119	33	167	36
**Total**	**213**		**248**		**44**		**51**		**366**		**461**	

**TABLE 5:** Nursing-related morbidity by age group.

Age group	Frequency		2013 and 2014		2014 and 2015		2015 and 2016	
	*n*	%	*n*	%	*n*	%	*n*	%
15–30	116	25	11	10	14	12	91	78
+30–45	122	27	12	10	13	11	97	79
+45–60	106	23	10	9	18	17	78	74
+60–93	117	25	11	9	6	5	100	86
**Total**	**461**		**44**		**51**		**366**	

A significant association was found between the disciplines (trauma and general surgery) and the nursing-related morbidities; *P* = 0.001. All of the nursing-related morbidities occurred more frequently in general surgery patients as compared to trauma patients. It was only adjunct management of nursing-related morbidity that was more common in the discipline of trauma.

### Nursing-related morbidities per category

The most commonly documented nursing-related morbidity was drugs and/or medication error (*n* = 167, 36%) with the second most common being adjunct management (*n* = 130, 28%). The data were further divided to illustrate the specific categories as per Table 4.

Adjunct management: Intravenous lines accounted for 68 (52%) of the morbidities and this included both central and peripheral lines being left open and becoming a potential site for infection as well as peripheral line infiltration. This was followed by intercostal drains (ICD) and gastric tubes each accounting for 31 (24%) of the nursing-related morbidities.

Wound management errors: The majority of nursing-related morbidities in this category involved poor dressing care (*n* = 31, 42%), and this included dressings omitted, especially after being exposed during the ward rounds.

Patient management: Bed and/or pressure sores accounted for the majority (*n* = 23, 77%) and this was found to be caused by poor pressure part care, with two reported cases caused by a pelvic binder and hard collars.

Resuscitation: There were four resuscitation nursing-related morbidities documented during the study period. There were two cardiopulmonary resuscitation (CPR) errors where nurses lacked competency in doing effective CPR as well as failing to commence CPR prior to the arrival of the doctor. The other two morbidities were related to failing to appropriately use resuscitation equipment.

Vital signs: A total of 40 (70%) vital sign omissions were reported and these included post-operation vital signs and normal routine vital signs not being carried out as required.

Drug errors: The majority of nursing-related morbidities in this category were related to drug omission (*n* = 123, 74%) which typically included antibiotics, anticoagulant, analgesia and chronic medication omission.

## Discussion

### Overview of nursing-related morbidity

This is the first study to report nursing-related morbidity in the surgical service of Pietermaritzburg metropolitan complex. The incident rate of 3.7% of nursing-related morbidity is consistent with the estimated rate of 2.5% to 18.4% found by Wilson et al. (2012) in developing countries (including South Africa). Kong and Clarke (2016) reported an incident rate of 4.1% morbidities during a 5-year analysis of morbidity and mortality conferences in a South African trauma service. However, the incident rate of the current study is lower than the findings of 12.7% in the low- to middle-income countries revealed by Jha et al. (2013). The difference in methodologies used between this current study (retrospective) and the observational study by Jha et al. (2013) could partly explain the higher incidence rate reported. Authors have reported the retrospective review method as having limitations that can contribute to the underestimation of morbidities (Wilson et al. 2012). There was an increase in the number of documented nursing-related morbidities during November 2015 to October 2016. However, the reason for this is not clear but may be because of increased familiarity with the reporting system.

The majority of the patients experiencing nursing-related morbidities were general surgery patients as these were the majority of the patients who were admitted to the service during the study period. The results showed that more males than females experienced nursing-related morbidities and this is in keeping with the results of a study by Kong and Clarke (2016).

### Nursing-related morbidities per category

Drugs and/or medication morbidities were documented as being the most frequently occurring nursing-related morbidity. Duarte et al. (2015) also highlighted medication errors as the most common adverse event related to nursing care after reviewing 21 studies of adverse events. A global study by Jha et al. (2013) reported drug-related incidents as the second most common adverse events in the low- to middle-income countries, while it is the most common event in the high-income countries. Kalisch (2015) supported the findings of the current study by reporting medication omission as problematic in the hospitals in the US. Medication errors are reported to be a serious phenomenon that is not only financially costly but may also result in the death of a patient (Wananani et al. 2015).

The second most common nursing morbidity was related to adjunct management and the majority of these were owing to intravenous lines that were left open after use, a potential site for central line associated blood stream infection that could lead to sepsis and eventually death in some patients (Kornbau et al. 2015). Another adjunct-management-related morbidity was the kinked ICD that could lead to persistent haemo/pneumothorax. Kong et al. (2014) stated the kinking of the ICD tube to be the most common complication for ICDs. Nasogastric tube blockage was also noted to be among the prevalent adjunct management morbidities, resulting in poor nutritional support owing to delayed enteral feeding. Pereira et al. (2013) identified accidental removal and blockage as the main reasons for the loss of gastric tubes in the intensive care unit. The core reason for blockage of these tubes was associated with not using measures to prevent the blockage of the tube.

Poor wound management such as leaving the wound exposed for long periods of time results in the wound being infected, leading to a prolonged healing process and sepsis. Guo and DiPietro (2010) state that infection impairs the wound healing process by prolonging the inflammatory phase and if it continues for long the wound then becomes septic.

Regarding vital signs, the omission of abnormal vital signs that were not reported leads to serious preventable complications such as hypovolaemia, and respiratory and cardiac arrests. The result of the current study compares favourably with the findings of a study conducted in three Michigan Hospitals by Kalisch, Landstrom and Hinshaw (2009) where 14% of vital sign errors were reported.

Patient management accounted for 16% of nursing-related morbidities in the current study and the majority of these morbidities were related to bed and/or pressure sores which may prolong hospital stay and expose patients to infection as a result of skin breakdown. In Brazil, pressure sores were described as one of the most common adverse events (Mendesa et al. 2013) and in Spain, 19.5% of pressure sores were reported in a 2-year observational study (Manzano et al. 2014). Falls were accountable for 17% of the total nursing-related morbidity and a study in the US reported 20% of morbidity was related to falls (Lucero, Lake & Aiken 2010). In contrast, a Korean study by Kang, Kim and Lee (2014) reported a falls rate of 60.5% although this study cannot be truly comparable to the current study because this high rate was based on nurses’ perception and it also included different disciplines – internal medicine, gynaecology and so on.

The nursing morbidity related to resuscitation was low in the current study and this was supported by McInnes et al. (2012) who also reported low rates of CPR errors, as did Clarke et al. (2013).

## Recommendations

- These results highlight specific areas of concern to be addressed in the current nursing curriculum.
- Use of checklists for monitoring of routine activities in order to reduce omissions – a checklist for wound care, turning of patients and so on – that can be ticked off once the procedure has been done.
- The findings highlighted areas for further research, for example similar studies with a broad geographic scope (including district, regional, central and private hospitals) to allow for generalisation of the results as well as investigating the contributing factors (including gender) to nursing-related morbidities.

## Strengths and limitations

This study served to highlight a very important aspect of nursing and added to a limited body of nursing knowledge in South Africa. As this was a retrospective study, the quality of data documented may have affected the results. Information bias owing to documentation of data by different people and at different times was for sure a concern; however, in an attempt to address this issue an administrator (senior doctor) was tasked with checking the data on a weekly basis to ensure accuracy and completeness. Underreporting of morbidities in the first 2 years of the study could have occurred as the HEMR system had just been initiated. The study was conducted in one tertiary hospital. However, as facilities and resources are different in district, regional and central hospitals, these findings may not be generalisable.

## Conclusion

The study has identified the six most commonly documented nursing-related morbidities in the surgical service of a state hospital. The nursing-related morbidity regarding drug administration was the most common morbidity, followed by adjunct management. The findings of this study have served to highlight this as an area of importance for nursing and could provide direction for further research and educational initiatives.
